# Simultaneous hyperthermia-chemotherapy with controlled drug delivery using single-drug nanoparticles

**DOI:** 10.1038/srep24629

**Published:** 2016-04-22

**Authors:** Itaru Sato, Masanari Umemura, Kenji Mitsudo, Hidenobu Fukumura, Jeong-Hwan Kim, Yujiro Hoshino, Hideyuki Nakashima, Mitomu Kioi, Rina Nakakaji, Motohiko Sato, Takayuki Fujita, Utako Yokoyama, Satoshi Okumura, Hisashi Oshiro, Haruki Eguchi, Iwai Tohnai, Yoshihiro Ishikawa

**Affiliations:** 1 Cardiovascular Research Institute, Yokohama City University, Graduate School of Medicine, Yokohama, 236-0004, Japan; 2 Department of Oral and Maxillofacial Surgery, Yokohama City University Graduate School of Medicine, Yokohama, 236-0004, Japan; 3 Department of Orthopedic Surgery, Yokohama Touhoukai Hospital, Yokohama, 236-0031, Japan; 4 Department of Environment and Natural Sciences, Graduate School of Environment and Information Sciences, Yokohama National University, Yokohama, 240-8501, Japan; 5 Department of Physiology, Aichi Medical University, Nagakute, Aichi 480-1195, Japan; 6 Tsurumi University School of Dental Medicine, Tsurumi, 230-8501, Japan; 7 Department of Pathology, Jichi Medical Universityepartment of Pathology, Tochigi, 329-2111, Japan; 8 Advanced Applied Science Department, Research Laboratory, IHI Corporation, Yokohama, 235-8501, Japan

## Abstract

We previously investigated the utility of μ-oxo *N,N*′- bis(salicylidene)ethylenediamine iron (Fe(Salen)) nanoparticles as a new anti-cancer agent for magnet-guided delivery with anti-cancer activity. Fe(Salen) nanoparticles should rapidly heat up in an alternating magnetic field (AMF), and we hypothesized that these single-drug nanoparticles would be effective for combined hyperthermia-chemotherapy. Conventional hyperthermic particles are usually made of iron oxide, and thus cannot exhibit anti-cancer activity in the absence of an AMF. We found that Fe(Salen) nanoparticles induced apoptosis in cultured cancer cells, and that AMF exposure enhanced the apoptotic effect. Therefore, we evaluated the combined three-fold strategy, i.e., chemotherapy with Fe(Salen) nanoparticles, magnetically guided delivery of the nanoparticles to the tumor, and AMF-induced heating of the nanoparticles to induce local hyperthermia, in a rabbit model of tongue cancer. Intravenous administration of Fe(Salen) nanoparticles *per se* inhibited tumor growth before the other two modalities were applied. This inhibition was enhanced when a magnet was used to accumulate Fe(Salen) nanoparticles at the tongue. When an AMF was further applied (magnet-guided chemotherapy plus hyperthermia), the tumor masses were dramatically reduced. These results indicate that our strategy of combined hyperthermia-chemotherapy using Fe(Salen) nanoparticles specifically delivered with magnetic guidance represents a powerful new approach for cancer treatment.

Head and neck cancer is the sixth most common cancer, accounting for 3% of all cancers worldwide. The incidence of new patients is increasing, particularly in developing countries^1^,^2^. Tumors are located in the oral cavity in 48% of cases, and 90% of these are oral squamous cell carcinoma. Oral malignancy, including tongue cancer, is associated with severe morbidity and has a long-term survival of less than 50%, in spite of advances in the treatment of oral cancer by surgery, chemotherapy, and radiation. The survival ratio of patients remains very low, mainly due to lymph node metastasis^2^. Surgical removal of the cancer tissues is the gold standard, but involves various complications, such as dysphagia or dysarthria. Therefore, more effective treatment for oral cancer, with fewer complications, needs to be developed.

Hyperthermia is a promising cancer therapy, in which cancer tissues are exposed to a slightly raised temperature^3^,^4^, thereby increasing their susceptibility to radio- or chemo-therapy. The most commonly used heating method in clinical practice is capacitive heating with a radiofrequency electric field^5^. However, a major technical problem with this method is difficulty in heating the target tumor to the desired temperature without damaging the surrounding normal tissues. This is because the electromagnetic energy must be directed from an external source, and thus must penetrate through normal tissues, causing an unavoidable temperature gradient. Although other hyperthermia modalities, including radiofrequency ablation and ultrasound hyperthermia, have been widely developed^6^,^7^,^8^, the efficacy of these modalities is limited to the size and depth of tumors, and major disadvantages include the inability to precisely target a specific tumor site by directing heat exposure specifically to the tumor.

In order to overcome these challenges, magnetic nanoparticles have been developed^9^,^10^,^11^. Magnetic nanoparticles generate heat when exposed to an alternating magnetic field (AMF) as a result of hysteresis and relaxation losses, resulting in heat production^10^,^12^,^13^,^14^. Magnetic particles may be directly injected at the site of a tumor, or delivered to the site by a magnet, followed by AMF exposure to produce heat within the tumor, enabling specific thermal ablation in the target area. The particles are usually made of iron oxide (IO)^15^, and do not have anti-cancer activity *per se* in the absence of an AMF. Micelles containing IO particles and anti-cancer drugs have been investigated^16^, but drugs may be rendered therapeutically ineffective by heat-induced degradation.

The key objective of drug delivery is to increase the concentration of a therapeutic agent in the tumor, because this not only enhances the anti-cancer effect, but also reduces toxic side effects. In 1913, Paul Ehrlich proposed the classic concept of drug delivery by a carrier that would transport therapeutic drugs specifically to the target organ^17^. Since then, numerous drug delivery technologies have been developed to accomplish this objective, including micelles, liposomes, antibody drugs, affinity targeting, and macromolecular drug carriers. However, these technologies face difficulties due to the inherent instability of the carriers and limited delivery efficacy.

In order to overcome these problems, we have focused on novel magnetic nanoparticles consisting of μ-oxo *N,N*′-bis(salicylidene)ethylenediamine iron (Fe(Salen)). As we previously reported^18^, Fe(Salen) nanoparticles act as a novel nano-magnetic agent with direct anti-cancer activity. The magnetic property of Fe(Salen) enables simple drug delivery using a readily available permanent magnet. The anti-cancer activity is similar to that of cisplatin, and this agent exhibits potent cytotoxicity, presumably via production of reactive oxygen species (ROS) that cause DNA damage. Importantly, the anti-cancer property of Fe(Salen) nanoparticles is stable at temperatures up to around 100 °C. Their magnetic character is also stable during repeated heat exposures, suggesting that Fe(Salen) nanoparticles might be available for simultaneous chemotherapy and hyperthermic therapy. Such combination therapy is potentially very effective, since, for example, hyperthermia has been shown to enhance cellular uptake of anti-cancer drugs^19^.

In this study, we examined the feasibility and effectiveness of combined hyperthermia-chemotherapy with magnetically guided Fe(Salen) nanoparticles to treat tongue cancer in a rabbit model, because the tongue is readily accessible within the oral cavity, and thus it is easy to apply a magnet and an AMF. Our results indicate that this strategy is indeed highly effective.

## Results

### Fe(Salen) nanoparticles showed stable magnetic and anti-cancer properties when exposed to an AMF

**Figure 1:**
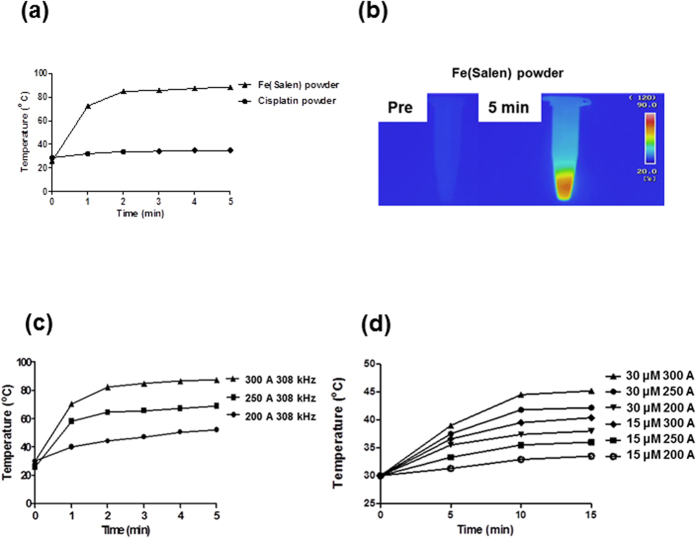
Heat is generated by Fe(Salen) nanoparticles upon exposure to an AMF. (**a**) Heat generation by cisplatin or Fe(Salen) upon AMF exposure. The AMF was applied at a frequency of 308 kHz and EC 250 A. (**b**) Representative thermography of Fe(Salen) nanoparticle dry powder in a tube before (*Pre*) and 5 min after AMF exposure (*5 min*). (**c**) Effect of an AMF (200, 250 and 300 A) on the temperature of Fe(Salen) nanoparticles in culture medium. (**d**) Effect of Fe(Salen) concentration (15 or 30 μM) and electrical current (200, 250 or 300 A) on the temperature in the culture medium.

Fe(Salen) is an iron-salen derivative that possesses both anti-cancer and intrinsic magnetic properties^18^. Previous measurements with a superconducting quantum interference device (SQUID) (Quantum Design MPMS7 system)^18^ suggested that Fe(Salen) nanoparticles could generate thermal energy due to hysteresis losses when exposed to an AMF. In the present study, we generated an AMF with a vertical coil, driven by a transistor inverter. In the dry powder state, the temperature of an AMF-exposed Fe(Salen) nanoparticle sample rose to above 80 °C within a few minutes (Fig. 1a,b). In contrast, no heat production occurred when cisplatin was used (Fig. 1a). When heat production was examined using an Fe(Salen) nanoparticle suspension in culture medium, the temperature increased to a lesser degree because of heat loss through the medium, but the temperature increase was both electric current (EC)-dependent (Fig. 1c) and concentration-dependent (Fig. 1d). For further experiments, we adopted AMF parameters that afforded a local temperature of 42 °C (frequency of 308 kHz and EC of 250 A) (Fig. 1c).

### Fe(Salen) nanoparticles exhibited anti-cancer effect on tongue cancer cells

**Figure 2:**
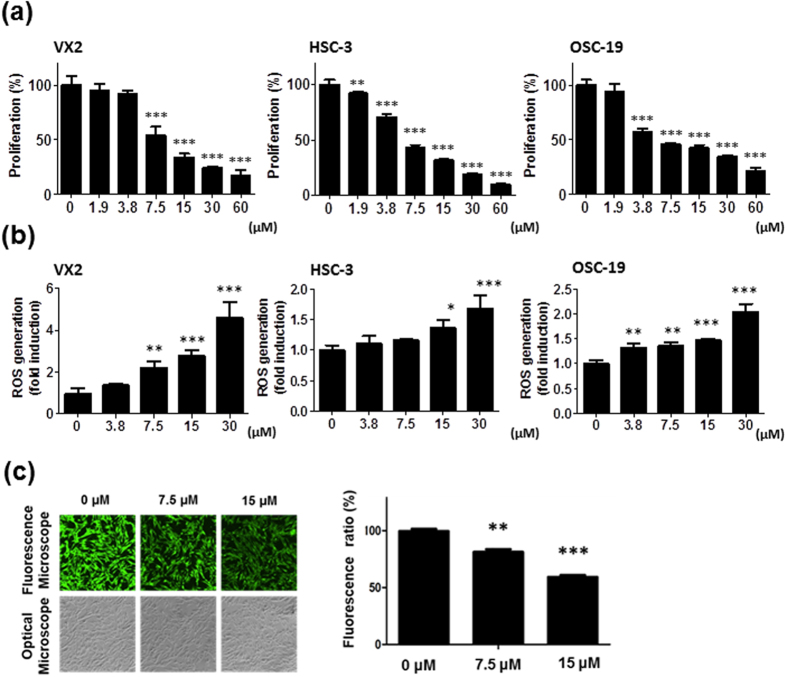
Fe(Salen) nanoparticles inhibit cell proliferation, promote ROS generation, and are taken up by cells. (**a**) Effect of Fe(Salen) on proliferation of various cancer cells. XTT cell proliferation assays were performed with human and rabbit tongue cancer cells: VX2 rabbit squamous cell carcinoma, HSC-3 human squamous cell carcinoma, and OSC-19 human squamous cell carcinoma (n = 4). The IC_50_ values were similar among cell types and were approximately 7.5 μM. (**b**) Effect of Fe(Salen) on ROS production in various cells. Fe(Salen) nanoparticles generated ROS in a concentration-dependent manner (n = 4, ^**^p < 0.01, ^***^p < 0.001). (**c**) Representative fluorescence pictures of calcein using a fluorescence microscope and optical microscope. Ratios of calcein fluorescence are shown below (n = 4, ^**^p < 0.01, ^***^p < 0.001 vs. control). Note that cellular fluorescence was decreased in the presence of Fe(Salen) nanoparticles.

Because tongue cancer cells are squamous in nature, we used various squamous cancer cell lines in culture, including VX2 (rabbit), HSC-3 (human), and OSC-19 (human). Fe(Salen) nanoparticles exhibited potent, dose-dependent anti-cancer effects on these cell types. The IC_50_ values were similar among these cell types (approximately 7 μM) (Fig. 2a). Next, we investigated the mechanism of cell death and the cellular uptake of Fe(Salen) nanoparticles. As we had previously demonstrated^18^, Fe(Salen) nanoparticles increased generation of reactive oxygen species (ROS) in a dose-dependent manner (Fig. 2b). We also examined the cellular uptake of Fe(Salen) nanoparticles using calcein (CA). CA is a fluorescent probe for cellular iron, which reflects the content of iron within mammalian cells^18^. When CA is bound to iron, the intensity of the fluorescence decreases within cells. When VX2 cells were incubated in the presence of Fe(Salen) nanoparticles, cellular fluorescence was decreased in a Fe(Salen) nanoparticle concentration-dependent manner (Fig. 2c), suggesting that Fe(Salen) nanoparticles were taken up, at least to some extent, by these cells.

### Cytotoxic and magnetic properties of Fe(Salen) nanoparticle were stable after heat exposure

**Figure 3:**
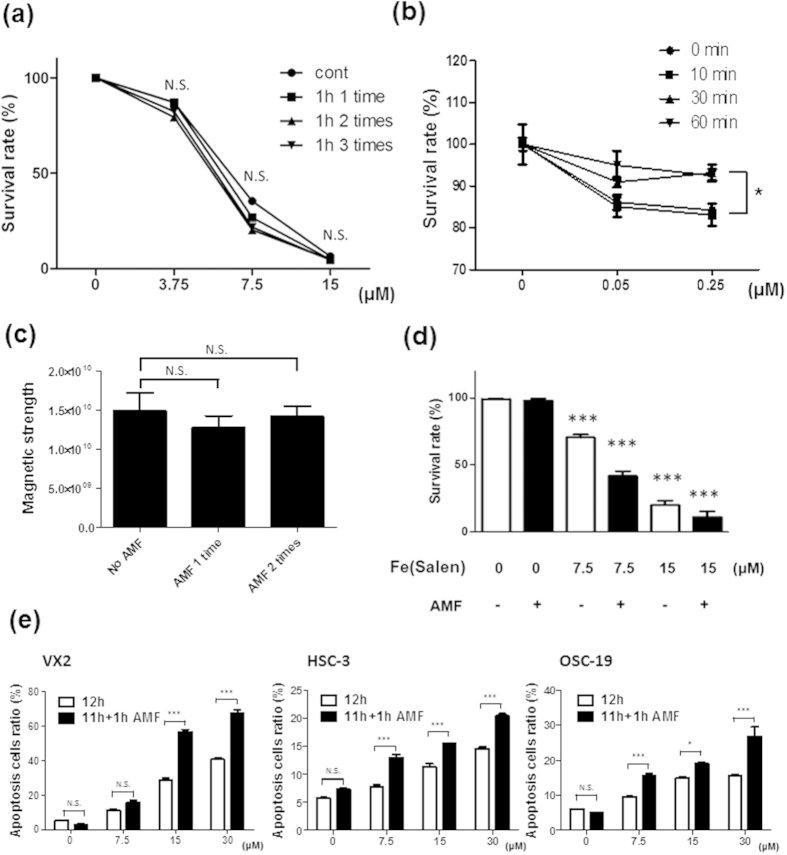
Fe(Salen) nanoparticle-induced apoptosis is increased by AMF exposure. (**a**) Effect of an AMF on Fe(Salen) nanoparticles. Fe(Salen) nanoparticles were heated to 80 °C by exposure to an AMF for 60 minutes once, twice, or three times. Changes in cytotoxic potency were examined in the presence of various concentrations of Fe(Salen) nanoparticles in VX2 cells. Note that there were no changes in cytotoxicity (n = 4, N.S., not significant). (**b**) Effect of high temperature (50 °C) on cytotoxic potency of Cetuximab (Erbitax^®^). Centuximab, a drug targeting epidermal growth factor receptor (EGFR), was heated to 50 °C for 30 or 60 minutes, followed by cytotoxicity assay in OSC-19 cells. Note that AMF exposure did not change the cytotoxicity of Fe(Salen) nanoparticles, but did change that of Cetuximab (Erbitax^®^) (n = 4, ^*^p < 0.05). (**c**) ESR analysis of magnetism after exposure to an AMF. *No AMF*; Fe(Salen) without AMF exposure, *AMF 1 time*; Fe(Salen) with AMF exposure for an hour once, *AMF 2 times*; Fe(Salen) with AMF exposure for one hour twice. (**d**) Increased anti-cancer effect of Fe(Salen) with AMF exposure. An AMF promoted cellular death of VX2 cells in the presence of Fe(Salen) nanoparticles, as determined by trypan blue staining (n = 4, ^**^p < 0.01, ^***^p < 0.001 vs. control). (**e**) Effect of Fe(Salen) with AMF exposure on various cancer cells. Apoptotic cell ratio is shown after incubation in the presence of various concentrations (0, 7.5, 15, 30 μM) of Fe(Salen) nanoparticles for 12 hours (*white bars*) or for 11 hours followed by 1 hour AMF exposure (*black bars*). Apoptosis was determined by Annexin-V/PI staining with FACS scan dot plot analysis at 12 hours after treatment with Fe(Salen) nanoparticles. Note that AMF exposure increased the cytotoxicity in the presence of Fe(Salen) nanoparticles (n = 4, ^*^p < 0.05).

We also examined the heat stability of Fe(Salen) *per se*, because many anti-cancer or micellar drugs are sensitive to high temperature and readily degraded. Fe(Salen) nanoparticles were heated to above 80 °C by AMF exposure for one hour. After the nanoparticles had cooled, they were added to cultured cancer cells. Fe(Salen) nanoparticles exhibited an identical cytotoxic effect after three cycles of heating-cooling to that of the unheated nanoparticles (Fig. 3a). This is most likely because the anti-cancer property is an intrinsic feature of the structurally stable Fe-Salen complex. In contrast, the cytotoxicity of Cetuximab (Erbitax^®^), which is an antibody drug targeting epidermal growth factor receptor (EGFR), decayed after 30 minutes at 50 °C in OSC-19 cells (Fig. 3b).

The magnetic properties also remained unchanged after exposure to heat. Even after AMF exposure for one hour twice, Fe(Salen) nanoparticles were similarly attracted by a magnet. We also confirmed the stability of the magnetism of Fe(Salen) nanoparticles by electron spin resonance (ESR) study (Fig. 3c).

Thus, the cytotoxic and magnetic properties of Fe(Salen) nanoparticle were both heat-stable, in contrast to the characteristics of conventional micellar drugs and anti-cancer drugs.

### Fe(Salen) nanoparticles were accumulated by a permanent magnet in cultured cells and mice

**Figure 4:**
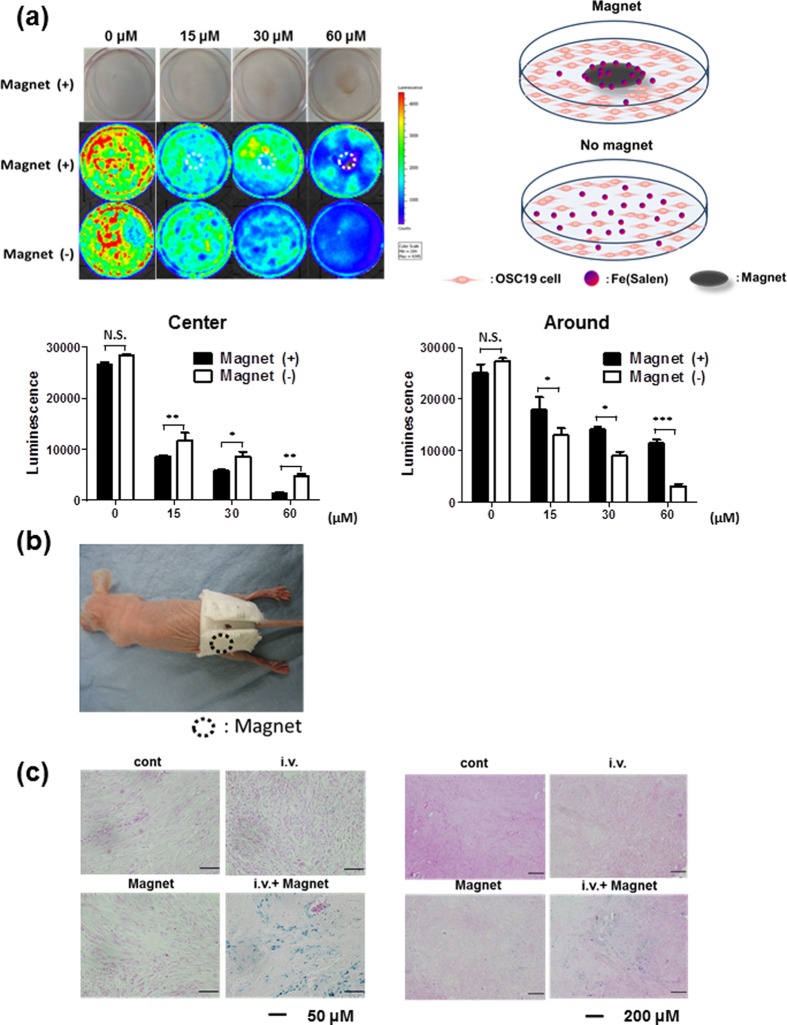
Fe(Salen) nanoparticles are attracted by a permanent magnet *in vitro* and in the mouse model *in vivo.* **(a)** Distribution of Fe(Salen) and cellular death in the presence of a magnet. Distributions of Fe(Salen) and cytotoxic effect were compared between the center (*center*), where the magnet was positioned, and the edge (*edge*) of each culture dish. *Upper photo*: distribution of various concentrations of Fe(Salen) in a dish with a magnet. *Middle photo*: Cell viability in the presence of a magnet. Cell viability was determined in terms of luciferase activity by intensity measurement with an IVIS imaging system. *Lower photo*: Cell viability in the absence of a magnet. Bar graphs show the determination of cell viability with IVIS (*center* and *edge*). (n = 4, ^***^p < 0.001) Note that the cytotoxicity of Fe(Salen) nanoparticles was enhanced in the center. In contrast, the cell viability at the edge of culture dishes is maintained in the presence of a magnet compared to that of in the absence of a magnet. **(b)** A Jacket used for drug delivery in mice. *Circle* shows the site where the permanent magnet is installed. (**c**) Representative photo of skin tissues at the site beneath the magnet in mice which had been injected with Fe(Salen) via the tail vein. Fe(Salen) was accumulated beneath the magnet at the tumor location. Mice were intravenously injected with Fe(Salen) (5 mg/kg) and wore a jacket for three days. Accumulation of Fe(Salen) nanoparticles was examined by Berlin blue staining. *Cont*; control, *iv*; Fe(Salen) was injected, *Magnet*; jacket was worn, *iv Magnet*; Fe(Salen) was injected and the jacket was worn. High (left, calibration bar 50 μm) and low magnification (right, 200 μm) images are shown.

We then examined the magnetic attraction of Fe(Salen) in cultured cells. Substantial accumulation of Fe(Salen) nanoparticles by a magnet was followed by enhanced local cytotoxicity. The cytotoxicity was strongest at the center of the culture dish, where the strength of the magnetic field was greatest (Fig. 4a).

Next, we examined magnetic attraction in a mouse model grafted with VX2 cells onto the legs. We built a custom jacket with a button-type permanent magnet (630 mT), which allowed the mouse to move freely (Fig. 4b). Fe(Salen) nanoparticles were injected via the tail vein, and the jacket was placed so that the button magnet was located on top of the leg tumor. After three days, we observed blue staining due to Fe(Salen) nanoparticles at the site below the magnet, confirming successful accumulation of Fe(Salen) nanoparticles by the permanent magnet *in vivo*. Such accumulation did not occur in the absence of the magnet (Fig. 4c).

### AMF exposure enhanced apoptosis induced by Fe(Salen) nanoparticles

We then examined the AMF-induced hyperthermic effect of Fe(Salen). An AMF enhanced the cytotoxic effect of Fe(Salen) nanoparticles on VX2 cells (Fig. 3d). Similar results were obtained when cellular apoptosis was examined by flow cytometry (Fig. 3e). We confirmed that an AMF *per se* had no effect in the absence of Fe(Salen). However, in the presence of Fe(Salen) nanoparticles, an AMF increased the number of apoptotic cells (Fig. 3e). In contrast, when cisplatin was used in place of Fe(Salen), an AMF had no effect on the rate of apoptosis or cell survival (*data not shown*), and there was no rise in temperature (Fig. 1a).

### Fe(Salen) nanoparticles generated heat, causing enhanced cytotoxicity in mice

**Figure 5:**
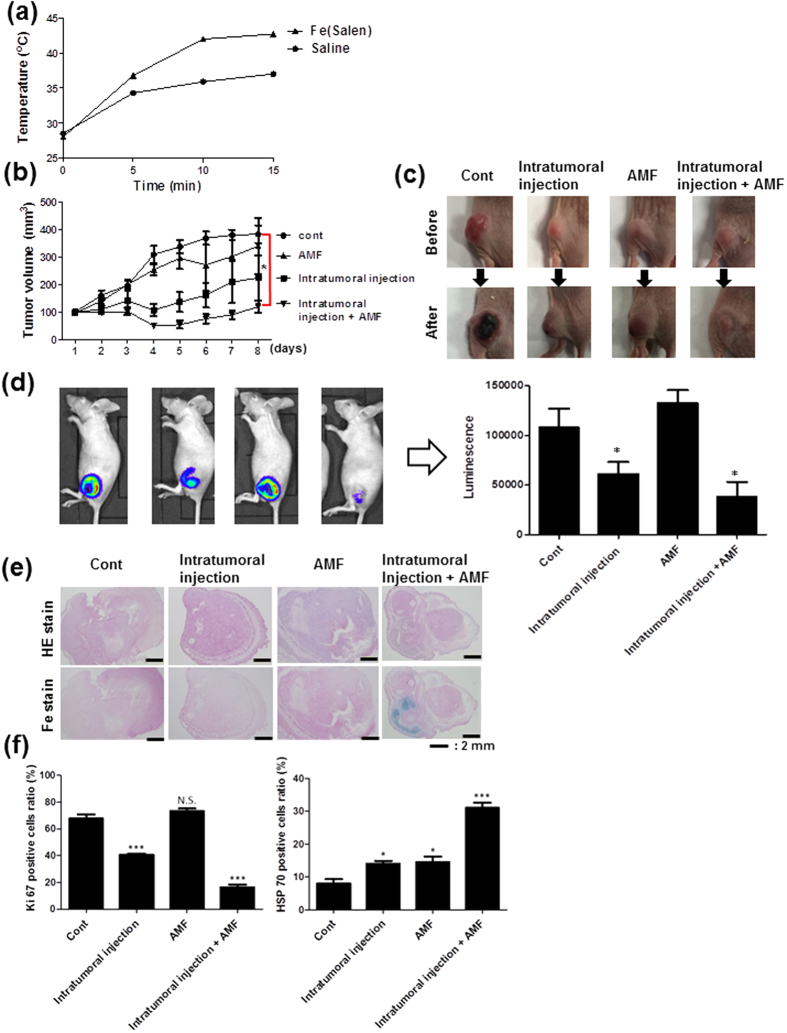
Local injection of Fe(Salen) nanoparticles generates heat upon exposure to an AMF; induced anti-cancer effect in a mouse tongue cancer model. **(a)** Changes in temperature with AMF exposure. Either Fe(Salen) nanoparticles or saline were injected into the tumor in mice, followed by exposure to an AMF. Note that the local temperature was increased to a greater degree with Fe(Salen) in a time-dependent manner. (**b**) Effect of Fe(Salen) nanoparticle injection and AMF exposure on tumor size. The graph showed the time course of tumor volume changes. Fe(Salen) inhibited tumor growth, and AMF exposure of Fe(Salen)-injected animals further inhibited the tumor growth. Mean tumor volume (mm^3^) of each group is also shown. Control (*cont*), intratumoral injection of Fe(Salen) nanoparticles (*injection*), AMF exposure alone (*AMF*), and intratumor injection of Fe(Salen) nanoparticles and AMF exposure (*injection and AMF*). (n = 6, ^*^p < 0.05, ^**^p < 0.01, ^***^p < 0.001). **(c)** Representative photo of tumors in each group. **(d)** Effect of Fe(Salen) nanoparticle injection and AMP exposure on tumor size, determined from IVIS images. Representative IVIS images of mouse tumors (*left*) and luminescence intensity in each group (*right*) are shown. **(e)** Representative photos of tumor tissues from each group. HE staining (*upper*) and Fe staining (*lower*) are shown. Calibration bar: 2 mm. (**f**) Expression of Ki67-positive cells (*left*) and HSP70-positive cells (*right*).

We then examined whether a tumor can be heated by an AMF after local injection of Fe(Salen) nanoparticles in mice. We implanted OSC-19 cells, human tongue cancer cells, on the back of mice to create an ectopic and heterologous human tongue cancer model. We used OSC-19 cells transfected with a luciferase-encoding vector for determination of the tumor size. Fe(Salen) nanoparticles (50 mM, one-third of the tumor volume per mouse) were locally injected at the tumor site, and the tumor was exposed to an AMF for 30 min twice a week. We found that the local temperature in the tumor was rapidly increased by the AMF, while that of saline increased only slowly (Fig. 5a). The tumor volume ratio was calculated every day. Tumors grew largest in the absence of Fe(Salen) nanoparticles (control; 382 ± 31.9% of the basal volume). Local injection of Fe(Salen) nanoparticles significantly reduced the tumor volume (223 ± 80.6%). When an AMF was applied, the tumor volume was further decreased at the same dose of Fe(Salen) (119 ± 21.9%). AMF exposure in the absence of the nanoparticles had no effect on the tumor volume (Fig. 5b,c).

Pathological examination by hematoxylin and eosin (HE) and by chemical iron staining revealed that Fe(Salen) nanoparticles remained at the site of local injection of Fe(Salen) after 8 days (Fig. 5e,f). Thus, AMF exposure after local injection of Fe(Salen) generated heat and effectively reduced the tumor volume in mice.

### Fe(Salen) nanoparticles were accumulated by a magnet, and produced heat upon exposure to an AMF in a rabbit oral cancer model

We next examined the combination of controlled drug delivery by a magnet and AMF-induced heat production in a rabbit model. We injected Fe(Salen) nanoparticles intravenously, not locally, into a rabbit tongue cancer model. For magnetic guidance, we used an electromagnet (EM) (EM-30200 V, Echo Electric Co. Yamanashi, Japan), which can generate 2 T magnetic field intensity (Supplementary Fig. 1a); this is sufficient to achieve a high level of controlled drug delivery within a short time. The magnetic field intensity was greatest at the center of the magnet, and elsewhere, the intensity was inversely proportional to the distance from the center (Supplementary Fig. 1b).

Fe(Salen) nanoparticles were intravenously injected, and the electromagnet was applied to the rabbit tongue tissue, followed by exposure to an AMF. Histological study showed that Fe(Salen) nanoparticles were accumulated by the electromagnet (Supplementary Fig. 1c). Measurement of the tongue temperature with a thermometer under anesthesia, showed that 10 minutes exposure to an AMF increased the temperature to 42 °C (Supplementary Fig. 1d).

**Figure 6:**
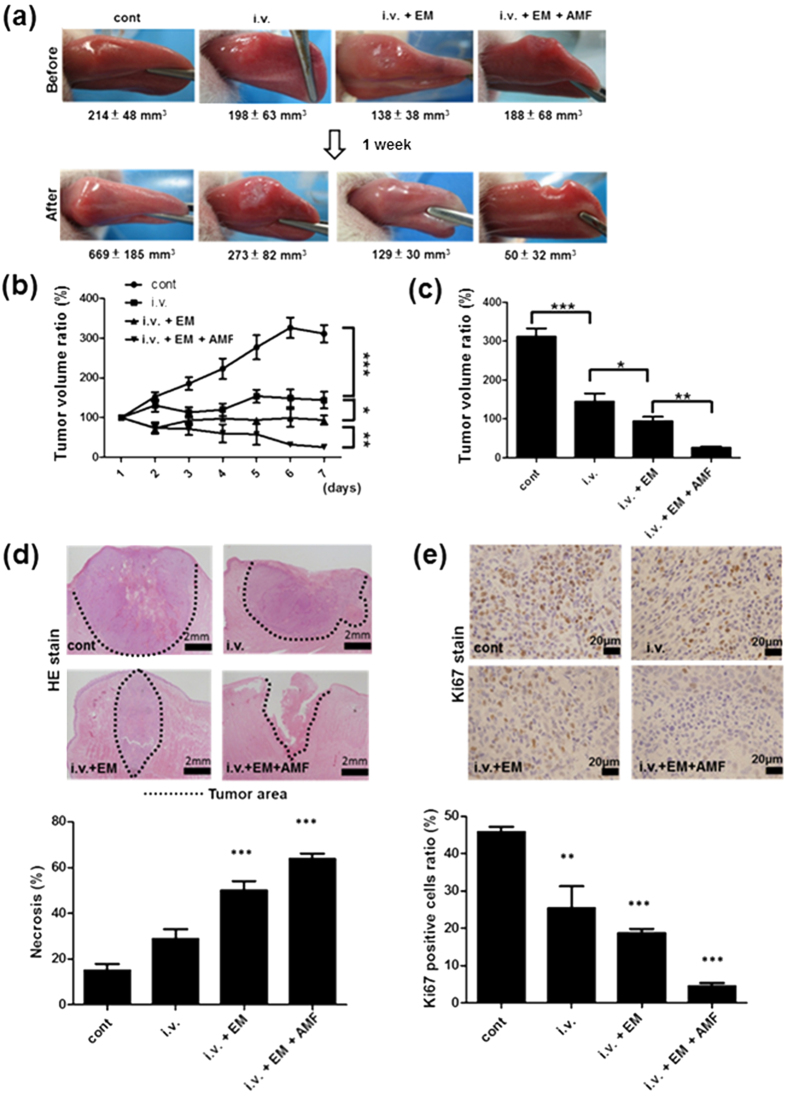
Anti-cancer effect of Fe(Salen) nanoparticles with magnet application and AMF exposure in rabbits. (**a**) Representative photos of rabbit tongue tumors in each group before (*upper*) and after (*lower*) treatment. Mean tumor volume value (mm^3^) of each group is also shown. Control (*cont*), intravenous injection of Fe(Salen) nanoparticles (*i.v*.), Fe(Salen) nanoparticle injection and electromagnet application (*i.v.* + *DDS*), and Fe(Salen) nanoparticle injection, electromagnet application, and AMF exposure (*i.v.* + *DDS* + *AMF*). (**b**) Changes in tumor volume ratio for 7 days. (n = 6, ^*^p < 0.05, ^*^^*^p < 0.01, ^*^^*^^*^p < 0.001). (**c**) Comparison of tumor volume ratios at day 7 (n = 6, ^*^p < 0.05, ^*^^*^p < 0.01,^*^^*^^*^p < 0.001). (**d**) Representative histological photo by HE staining at day 7 (*upper*). Broken lines indicate tumor areas. Calibration bar: 2 mm. Quantification of necrotic area by HE staining (*lower*) (n = 4, ^*^^*^^*^p < 0.001). (**e**) Representative pictures of tongue tumors after Ki67 staining (*upper*). Calibration bar: 20 μm. Quantification of necrosis by Ki67 staining in rabbit tongue tumors (*lower*) (n = 4, ^*^^*^p < 0.01, ^*^^*^^*^p < 0.001).

Tumors grew largest in the absence of Fe(Salen) nanoparticles (control: 311.3 ± 53.2% of the basal volume). Intravenous injection (i.v.) of Fe(Salen) nanoparticles (5 mg/kg) every day for 7 days significantly reduced the tumor size (144.3 ± 51.3%: *i.v. group*). When the electromagnet was applied to the tumor site immediately after the intravenous injection, the same dose of Fe(Salen) further decreased the tumor volume (93.8 ± 27.9%; *i.v.* + *EM group*). When an AMF was applied after the electromagnet, the tongue tumor almost completely disappeared after 1 week (25.7 ± 8.4%: *i.v.* + *EM* + *AMF group*) (Fig. 6a–c). There was no increase in kidney or liver enzymes in any of the groups at this dose level (Supplementary Fig. 4).

The VX2 carcinoma is a rapidly growing tumor, which has an ischemic center that subsequently undergoes necrosis, but has a well vascularized periphery^20^. Pathological examination by HE (Fig. 6d) and Ki67 staining (Fig. 6e) revealed that Fe(Salen) nanoparticles augmented tumor tissue death. In the *control group*, the area of necrosis was the smallest (15.0 ± 5.8%); it was increased in the *i.v. group* (28.8 ± 8.5%), further increased in the *i.v.* + *EM group* (50.0 ± 8.2%), and was greatest in the *i.v.* + *EM* + *AMF group* (63.8 ± 4.8%). Concentrations of Ki67-positive cells were also changed accordingly (*control*, 45.8 ± 2.7%; *i.v.*, 25.4 ± 11.8%; *i.v.* + *EM*, 18.8 ± 2.2%; *i.v.* + *EM* + *AMF*, 4.5 ± 1.7%). IVIS imaging confirmed that EGFP signal was the lowest in *i.v.* + *EM* + *AMF* group (Supplementary Fig. 3). These results indicate that both application of the electromagnet and exposure to an AMF greatly enhance the anti-cancer effect of Fe(Salen) nanoparticles.

## Discussion

In the current study, we demonstrated that the three-fold strategy of chemotherapy with Fe(Salen) nanoparticles, magnetically guided delivery of the nanoparticles to the tumor, and AMF-induced heating of the nanoparticles to induce local hyperthermia exhibited a potent anti-cancer effect in a rabbit model of tongue cancer *in vivo*. It is noteworthy that a high drug dose was not necessary for successful results in either application of the electromagnet or AMF exposure. We used an electromagnet instead of a permanent magnet to induce hyperthermia in this model, because a permanent magnet was difficult to apply onto the tongue surface for a long time. Nevertheless, it is noteworthy that a simple permanent magnet was effective in the leg tumor model. Thus, in future trial with tumors of the extremities or the trunk, it should be possible to accumulate Fe(Salen) nanoparticles sufficiently by using a simple jacket equipped with a small permanent magnet.

The anti-cancer and magnetic properties of Fe(Salen) nanoparticle were unaffected by AMF exposure, in marked contrast to conventional micelle-based drugs containing magnetic particles and anticancer drugs or antibody drugs, which are readily degraded and lose their potency upon exposure to heat. In particular, conventional inorganic particles lack intrinsic anti-cancer effects, and so additional procedures to transport/conjugate drug compounds are required^16^. Notably, the use of Fe(Salen) nanoparticles enables anticancer and hyperthermic therapies to be performed simultaneously, and specifically at the cancer site; this is a major advantage compared to conventional hyperthermal approaches.

Some issues remain before clinical application will be possible. Firstly, Fe(Salen) nanoparticles as a drug compound are still at non-GMP (Good Manufacturing Practice) status at this time. Thus, we should establish a protocol for synthesis of Fe(Salen) nanoparticles with appropriate magnetic properties, particle size, and purity to meet GMP standards for human use. Our elemental analysis and Fourier-transform infrared spectroscopy (FT-IR) analysis of Fe(Salen) samples^18^ indicated that they are highly pure (>95%) and do not contain metals other than iron that may potentially exhibit magnetism or cytotoxicity. Secondly, the origin of the magnetic properties needs further investigation. Although Fe(Salen) possess a crystal structure that accords with the classical Goodenough-Kanamori-Anderson rule^21^ regarding magnetic interactions^18^, we do not know whether this is necessary and sufficient. Further collaborative investigation with physicists may be necessary in order to develop future drug compounds for magnetically guided delivery. Thirdly, therapeutic protocols for hyperthermia need to be optimized to for human patients. The current protocol applies hyperthermic therapy between bouts of minimally invasive chemotherapy. It may be desirable to develop a more effective AMF generator or coil to optimize the efficacy of drug delivery and hyperthermia.

In the current study, we have established that Fe(Salen) nanoparticles are highly effective for treatment of tongue cancer in a rabbit model. Fe(Salen) may also be suitable for magnetization of existing anti-cancer drugs, as we have recently demonstrated by synthesizing a magnetized derivative of paclitaxel (Umemura *et al. unpublished observations*). It should be possible to extend this approach to other widely used anti-cancer drugs to enable magnetically controlled delivery and/or hyperthermic therapy, and such an approach is expected to offer increased therapeutic efficacy with reduced side effects, in principle. Such agents would be promising tools for “on-demand” cancer therapy^22^, using unique metal ligand complex-based anti-cancer drugs with intrinsic magnetic properties to target specific cancers in a highly controllable manner.

## Methods

### Reagent and cell culture

Fe(Salen) nanoparticle reagent was purchased from Tokyo Chemical Industry Co. Ltd. Fe(Salen) was sonicated for 30 min and used in normal saline suspension as previously described^18^. The average size of nanoparticles was about 200 nm. Cetuximab (Erbitax^®^) was purchased from Merck Serono (Tokyo, Japan). Rabbit squamous cell carcinoma (VX2) cells were purchased from American Type Culture Collection (ATCC) (Virginia, U.S.A.). VX2 cells were transfected with an EGFP-encoding vector as previously described^23^. Human oral squamous cell carcinoma cell lines, OSC-19 and HSC-3, were purchased from the Health Science Research Resources Bank (Japan Health Sciences Foundation, Tokyo, Japan). In all cases, early passage cultures were stored and used for experiments. These cell lines were cultured in RPMI-1640 with L-glutamine and phenol red medium containing 10% fetal bovine serum (FBS) and 1% penicillin-streptomycin.

### Electromagnet device

We used an electromagnet (EM-30200 V) of 20 mm in diameter (Echo Electric Co., Yamanashi, Japan), capable of generating a 2 T field (Supplementary Fig. 1a,b).

### Alternating magnetic field (AMF) generator

An AMF was generated by a vertical coil with an inner diameter of 4 cm, driven by a transistor inverter (Hot Shot, Ameritherm Inc., New York, U.S.A.). Experiments were performed at a frequency of 308 kHz and EC 250 A unless otherwise specified^19^,^24^.

### Thermometer and thermography

A thermometer (hand-held thermometer HA-200, Anritsu Meter Co., Tokyo Japan) or a thermograph (InfraRed camera, Nippon Avionics Co., Ltd., Tokyo, Japan) was used to determine temperature *in vivo* and *in vitro*^19^.

### Sodium 2,3,-bis(2-methoxy-4-nitro-5-sulfophenyl)-5-[(phenylamino)-carbonyl]-2 H-tetrazolium inner salt (XTT) assay

Cell proliferation assay was performed using a commercially available kit, XTT Cell Proliferation Assay Kit (ATCC, Virginia, U.S.A), according to the manufacturer’s protocols^19^. VX2, HSC-3 and OSC-19 cells were seeded into each well of 96-well plates. The inoculated plates were incubated at 37 °C for 3 hours in an atmosphere of 5% CO_2_ in humidified air. Blank control wells contained medium without Fe(Salen) nanoparticles. Cells were then incubated with 5% CO_2_ at 37 °C in the presence of FeS for 24 hours. After incubation, Activated-XTT Solution was added to each well, and the plates were returned the incubator for 3 hours. The wells containing the cells and blank controls were measured with a microplate reader.

### Measurement of reactive oxygen species (ROS)

Measurement of ROS was performed as we previously reported^25^. VX2 cells were plated in 96-well culture plates overnight. Cells were then treated with 0, 3.6, 7.5, 15, and 30 μM Fe(Salen) nanoparticle at 37 °C for 24 hours. Intracellular ROS level was then measured using a fluorescent dye, 2′,7′-dichlorofluorescein diacetate (DCFH-DA; Sigma, Japan)^26^. In the presence of oxidant, DCFH is converted into highly fluorescent 2′,7′-dichlorofluorescein. Cells were first washed with PBS, and serum-free Minimum Essential Medium (MEM) containing DCFH-DA was added to each well. Cells were then incubated at 37 °C for 40 min. ROS production was measured using a microplate reader equipped with a spectrofluorometer (ARVO-Mx, PerkinElmer, Massachusetts, U.S.A.) at an emission wavelength of 538 nm and extinction wavelength of 485 nm.

### Calcein assay

Calcein-AM was purchased from Sigma^27^. VX2 cells in medium were seeded on each well of two 24-well plates. Cells were incubated for 24 hours. RPMI-1640 medium was changed to serum-free medium, followed by addition of 1 μM calcein and incubation at 37 °C for 1 hour. The medium was changed to RPMI-1640 containing L-glutamine, phenol red, 10% FBS, and 1% penicillin-streptomycin, and then Fe(Salen) nanoparticles (0, 7.5 and 30 μM) were added at room temperature in darkness. The cells were further incubated at room temperature for 3 hours. RFU was measured using a microplate reader equipped with a spectrofluorometer (ARVO-Mx) at an emission wavelength of 535 nm and extinction wavelength of 485 nm. Pictures were then taken with a fluorescence microscope.

### Magnet-guided delivery of Fe(Salen) nanoparticles *in vitro*

VX2 cells (6 × 10^4^ cells) were seeded in RPMI-1640 with L-glutamine and phenol red medium. Cells were incubated for 24 hours. Fe(Salen) nanoparticle suspension was added to the samples and adjusted to the concentration of 0, 3.7, 7.5, or 15 μM. VX2 cells were incubated with/without a permanent magnet at the center of each dish at 37 °C in 5% CO_2_ for 24 hours. The cells were fixed with formalin for 30 min at room temperature, and washed twice with PBS. Samples were evaluated by staining with hematoxylin and eosin (HE). Staining at the center and side of the samples was compared.

### Apoptosis assays

Apoptosis assays were performed as previously described^19^. VX2 cells were seeded on 6 cm dishes, and incubated for 24 hours. Fe(Salen) nanoparticles was then added to give 7.5, 15 or 30 μM, and incubation was continued for 12 hours. Cells were washed twice with cold PBS, and transferred into culture tubes. APC Annexin V and 7-AAD (BD Biosciences, California, U.S.A.) were then added to the tubes. Cells were incubated for 15 min at RT (25 °C) in darkness, followed by measurement with a FACS Canto™ II (Japan Becton, Dickinson and Company, Tokyo, Japan) within 1 hour.

### Intratumoral injection of Fe(Salen) nanoparticles in a mouse model implanted with human oral cancer and exposure to AMF

OSC-19 cells that had been transfected with luciferase-encoding vector were implanted into the back of mice to create a human oral cancer model. The tumors were allowed to grow to a size of 7–10 mm, and then Fe(Salen) nanoparticles (50 mM, one-third of the tumor volume per mouse) were locally injected into the tumor and the human tongue tissue was exposed to AMF at a frequency of 308 kHz and EC 300 A for 30 min twice a week. The tumor volume ratio was calculated by dividing the volume of each tumor by the baseline volume every day. After 8 days, tumors were harvested, and pathological examination was performed by HE and chemical iron staining. Immuno-histochemical examinations were also performed by staining with Ki67 and HSP70 antibodies. Evaluations were performed by two pathologists in a double-blind fashion.

### Magnetically controlled delivery of Fe(Salen) nanoparticles and hyperthermic therapy in tumor-bearing rabbits

Rabbits bearing tongue tumors were generated as previously described^28^,^29^. Male Japanese white rabbits weighing 2.0–2.5 kg were purchased from Nihon SLC (Shizuoka, Japan). Under general anesthesia, we implanted VX2 cells transfected with an Enhanced Green Fluorescent Protein (EGFP)-encoding vector in the right edge of the tongue to create a rabbit tongue cancer model^23^,^28^,^29^, in which to examine the effect of Fe(Salen), the electromagnet, and AMF. The tumors were allowed to grow to a size of 7–10 mm in length (usually for 7 days) prior to treatment. The rabbits (n = 24) were then separated into 4 groups: control (*control group*), intravenous Fe(Salen) injection (5 mg/kg) (*i.v. group*), intravenous Fe(Salen) nanoparticle injection (5 mg/kg) with electromagnet application (*i.v.* + *EM group*), and intravenous Fe(Salen) nanoparticle injection (5 mg/kg) with both electromagnet application and AMF exposure (*i.v.* + *EM* + *AMF group*). Fe(Salen) nanoparticles (5 mg/kg) were injected slowly via an ear vein once a day. For the electromagnet application, an EM-30200 V electromagnet (2 T), was positioned at the tongue tumor for 1 hour immediately after Fe(Salen) nanoparticle injection. These processes were repeated for 7 days. For AMF exposure, the rabbit tongue was inserted into a vertical coil and exposed to an AMF (308 kHz and EC 250 A) for 1 hour after the electromagnet application, twice a week. After 7 days, the tumors were harvested and evaluated by pathological examination, HE and Ki67 staining. Evaluations were performed by two pathologists, in a double-blind manner.

Animal experiments were performed according to the Yokohama City University guidelines for experimental animals. The Animal Care and Use Committee at Yokohama City University, School of Medicine, approved all animal studies.

### Evaluation of anti-cancer effects

The size of the tumors was measured daily under general anesthesia for 7 days. Tumor volume was determined using the following formula:


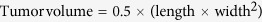


After the completion of the experiment, rabbits were sacrificed, and the tongues were harvested for histological evaluation by HE, Ki67 and Berlin blue staining (for Fe(Salen)).

### Data analysis and statistics

Statistical comparisons among groups were performed using Students’ *t*-test or one-factor analysis of variance (ANOVA) with the Bonferroni post hoc test. Statistical significance was set at the 0.05 level. Histological comparison was performed using the Kruskal-Wallis test (Fig. 5f 6d and e).

## Additional Information

**How to cite this article**: Sato, I. *et al*. Simultaneous hyperthermia-chemotherapy with controlled drug delivery using single-drug nanoparticles. *Sci. Rep.* **6**, 24629; doi: 10.1038/srep24629 (2016).

## Supplementary Material

Supplementary Information

## References

[b1] Aguzzi A. . MAP kinase modulation in squamous cell carcinoma of the oral cavity. Anticancer Res 29, 303–308 (2009). 19331166

[b2] Tanaka T., Tanaka M. & Tanaka T. Oral carcinogenesis and oral cancer chemoprevention: A Review. Patholog Res Int 2011, 10 (2011). 10.4061/2011/431246 PMC3108384 21660266

[b3] Radiofrequency Hyperthermia for Cancer. Lancet 323, 885–886 (1984). 6143190

[b4] Suit H. D. & Shwayder M. Hyperthermia: Potential as an anti-tumor agent. Cancer 34, 122–129 (1974). 4599460 10.1002/1097-0142(197407)34:1<122::aid-cncr2820340118>3.0.co;2-r

[b5] Abe M. H. . Multi-institutional studies on hyperthermia using an 8-MHz radiofrequency capacitive heating device (Thermotron RF-8) in combination with radiation for cancer therapy. Cancer 58, 1589–1595 (1986). 3756783 10.1002/1097-0142(19861015)58:8<1589::aid-cncr2820580802>3.0.co;2-b

[b6] Oura S. T. . Radiofrequency ablation therapy in patients with breast cancers two centimeters or less in size. Breast cancer (Tokyo, Japan) 14, 48–54 (2007). 10.2325/jbcs.14.48 17244994

[b7] Mardynskii Iu S. L. & V. F. Bizer V. A. Local ultrasound hyperthermia as a component of radiotherapy for osteogenic sarcoma of tubular bones in children and adolescents. Vopr onkol 53, 584–588 (2007). 18154126

[b8] Chatterjee D. K. & Krishnan S. D. P. Nanoparticle-mediated thermia in cancer therapy. Ther Deliv 2, 1001–1024 (2011). 22506095 10.4155/tde.11.72 PMC3323111

[b9] Jordan A. W., Fahling P., John H., Hinz W. & Felix A.R. Inductive heating of ferrimagnetic particles and magnetic fluids: physical evaluation of their potential for hyperthermia. Int J Hyperthermia 9, 51–68 (1993). 8433026 10.3109/02656739309061478

[b10] Minamimura T. S. . Tumor regression by inductive hyperthermia combined with hepatic embolization using dextran magnetite-incorporated microspheres in rats. Int J Onco 16, 1153–1158 (2000). 10.3892/ijo.16.6.1153 10811989

[b11] Maier-Hauff K. . Intracranial Thermotherapy using magnetic nanoparticles combined with external beam radiotherapy: Results of a feasibility study on patients with glioblastoma multiforme. J Neurooncol 81, 53–60 (2007). 16773216 10.1007/s11060-006-9195-0

[b12] Wang X. . Contribution of a 300 kHz alternating magnetic field on magnetic hyperthermia treatment of HepG2 cells. Bioelectromagnetics 34, 95–103 (2013). 23059525 10.1002/bem.21761

[b13] Atsumi T. J. Balachandran.Sato, Yoshinori.Tohji, Kazuyuki. Heating efficiency of magnetite particles exposed to AC magnetic field. J Mag Mag Mater 310, 2841–2843 (2007).

[b14] Thiesen B. & Jordan A. Clinical applications of magnetic nanoparticles for hyperthermia. Int J Hyperthermia 24, 467–474 (2008). 18608593 10.1080/02656730802104757

[b15] Qun Zhao L. W. . Magnetic Nanoparticle-based hyperthermia for head & neck cancer in mouse models Theranostics 2, 113–121 (2012). 22287991 10.7150/thno.3854 PMC3267386

[b16] Panagiotopoulos N D. R. . Magnetic particle imaging: current developments and future directions. Int J Nanomedicine 2015, 3098–3114 (2015). 10.2147/IJN.S70488 PMC4411024 25960650

[b17] Ehrlich P. Address in Pathology, On chmotherapy: Delivered before the seventeenth international congress of medicine. Br Med J 2, 353–359 (1913). 20766753 10.1136/bmj.2.2746.353 PMC2345634

[b18] Eguchi H. . A magnetic anti-cancer compound for magnet-guided delivery and magnetic resonance imaging. Sci. Rep. 5, 9194 (2015). 25779357 10.1038/srep09194 PMC4361848

[b19] Sato I. . Hyperthermia generated with ferucarbotran (Resovist®) in an alternating magnetic field enhances cisplatin-induced apoptosis of cultured human oral cancer cells. J Physiol Sci 64, 177–183 (2014). 24619404 10.1007/s12576-014-0309-8 PMC10717732

[b20] Jefferis AF B. M. The rabbit VX2 tumour as a model for carcinomas of the tongue and larynx. Acta Otolaryngol 108, 152–160 (1989). 2763834 10.3109/00016488909107407

[b21] Goodenough J. B. Theory of the role of covalence in the perovskite-type manganites [La, M(II)]MnO_3_. Phys Rev 100, 564–573 (1955).

[b22] Kuroda K., Okumura K., Isogai H. & Isogai E. The human cathelicidin antimicrobial peptide LL-37 and mimics are potential anticancer drugs. Front Oncolog 5, 144, doi: 10.3389/fonc.2015.00144 (2015). PMC4485164 26175965

[b23] Oshiro H. . Establishment of successively transplantable rabbit VX2 cancer cells that express enhanced green fluorescent protein. Med Mol Morphol 48, 13–23 (2014). 24573404 10.1007/s00795-014-0071-2

[b24] Hayashi K N. M. . Superparamagnetic nanoparticle clusters for cancer theranostics combining magnetic resonance imaging and hyperthermia treatment. Theranostics 3, 366–376 (2013). 23781284 10.7150/thno.5860 PMC3677408

[b25] Fukumura H. . Effect of ascorbic acid on reactive oxygen species production in chemotherapy and hyperthermia in prostate cancer cells. J Physiol Sci 62, 251–257 (2012). 22392350 10.1007/s12576-012-0204-0 PMC10717908

[b26] Setsukinai K., Urano Y., Kakinuma K., Majima H. J. & Nagano T. Development of novel fluorescence probes that can reliably detect reactive oxygen species and distinguish specific species. J Bio Chem 278, 3170–3175 (2003). 12419811 10.1074/jbc.M209264200

[b27] Gaboriau F. . Effects of deferasirox and deferiprone on cellular iron load in the human hepatoma cell line HepaRG. BioMetals 23, 231–245 (2010). 19997770 10.1007/s10534-009-9281-9

[b28] Hamaguchi S. . Selective hyperthermia using magnetoliposomes to target cervical lymph node metastasis in a rabbit tongue tumor model. Cancer Sci 94, 834–839 (2003). 12967484 10.1111/j.1349-7006.2003.tb01527.x PMC11160162

[b29] Dünne A. A. . Intravenous chemotherapy with cisplatin for regional lymph node metastases of auricular VX2 carcinoma. Anticancer Res 24, 1785–1790 (2004). 15274356

