# Supplementary material for: Simultaneous hyperthermia-chemotherapy with controlled drug delivery using single-drug nanoparticles
Source: Sci Rep. 2016 Apr 22;6:24629. doi: 10.1038/srep24629 (PMC4840378; doi:10.1038/srep24629)
Supplement: Supplementary Information [file srep24629-s1.doc]

Supplementary Materials for

**Simultaneous hyperthermia-chemotherapy with controlled drug delivery using single-drug nanoparticles**

Itaru Sato, Masanari Umemura, Kenji Mitsudo, Hidenobu Fukumura, Jeong-Hwan Kim, Yujiro Hoshino Hideyuki Nakashima, Mitomu Kioi, Rina Nakakaji, Motohiko Sato, Takayuki Fujita, Utako Yokoyama, Satoshi Okumura, Hisashi Oshiro, Haruki Eguchi, Iwai Tohnai and Yoshihiro Ishikawa

**This file includes:**

Supplementary Text

Figures S1 to S4

**
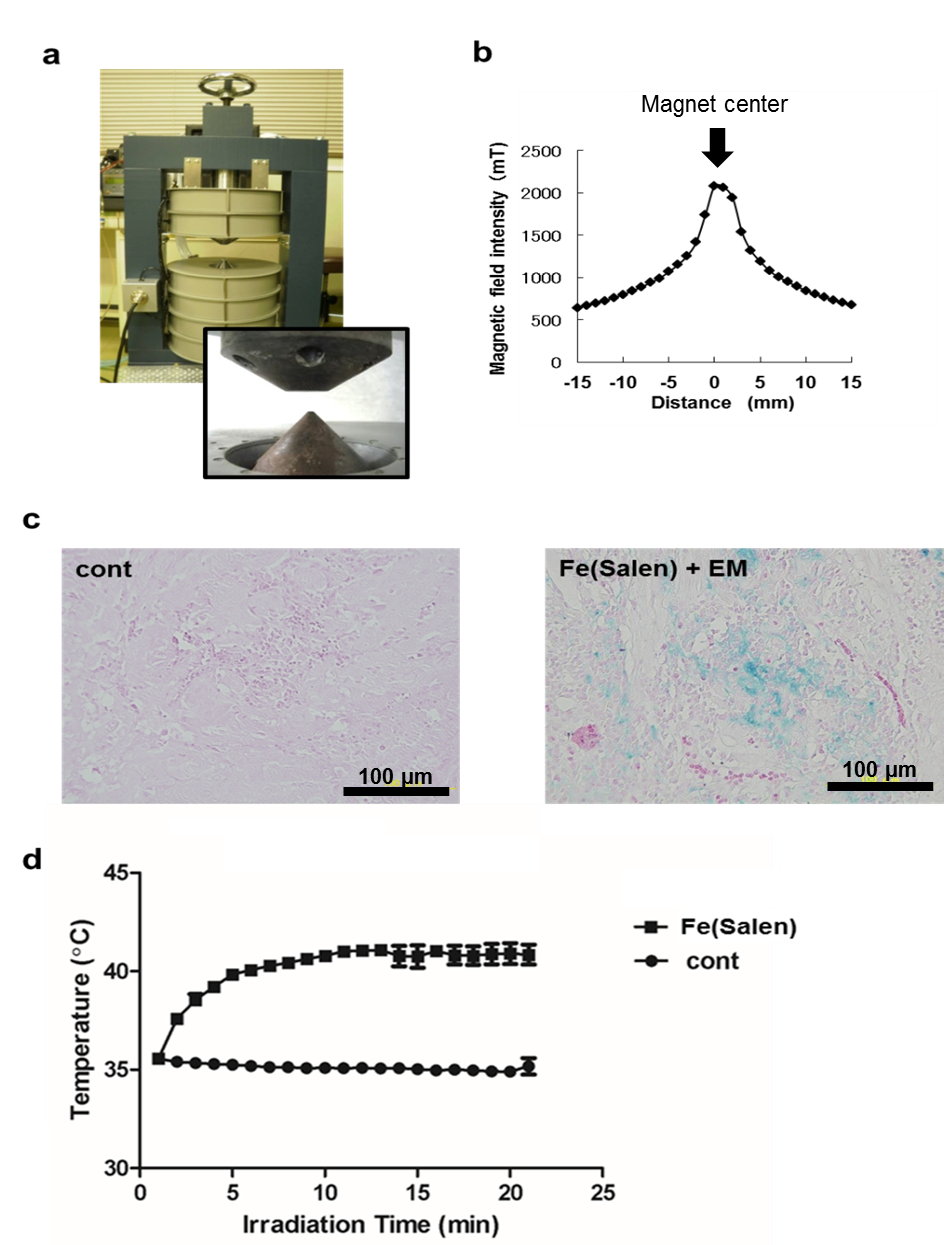
**

**Supplementary Figure 1 In a rabbit oral cancer model, Fe(Salen)** **nanoparticles are attracted by an electromagnet and generate heat in an AMF**

(**a**) An electromagnet (EM-30200V) was used to attract Fe(Salen) nanoparticles. The enlarged view indicates the center of the magnet. (**b**) Magnetic field intensity was greatest at the magnetic center, and decreased towards the periphery in a manner that was inversely proportional to the distance from the center. (**c**) Attraction of Fe(Salen) nanoparticles to rabbit tongue tumor tissue by the electromagnet (see also **Fig. 6**) was demonstrated by chemical iron staining (*left*, control; *right*, i.v. Fe(Salen) nanoparticle injection, followed by electromagnet application (EM)) (scale bar 100 μm).

(**d**) Heat production by Fe(Salen) nanoparticles/EM/AMF in rabbit tongue *in vivo*. Rabbits were intravenously injected with Fe(Salen) nanoparticles and the electromagnet was applied to the tongue. An AMF was then applied, and the tongue temperature was measured with a thermometer under anesthesia. Note that the temperature increased to 42 °C within 10 minutes (n=4).

**
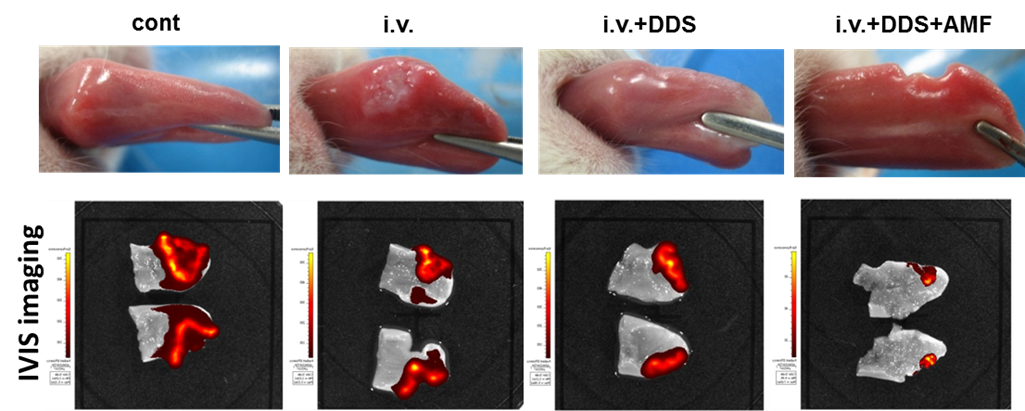
**

**Supplementary Figure 2. Representative RFP signals of rabbit tongue tumors in IVIS imaging.**

After completion of the *in vivo* experiment, rabbits were sacrificed, and the tongues were harvested for IVIS imaging to evaluate the size of the tumors. IVIS imaging was performed using rabbit tongue tumor specimens (see Fig. 6) after 7 days *ex vivo*. (**a**) control (*cont*), (**b**) intravenous injection of Fe(Salen) (*i.v*.), (**c**) Fe(Salen) nanoparticle injection and electromagnet application (*i.v.+DDS*), (**d**) Fe(Salen) nanoparticle injection, EM, and AMF application (*i.v.+DDS+AMF*).

**
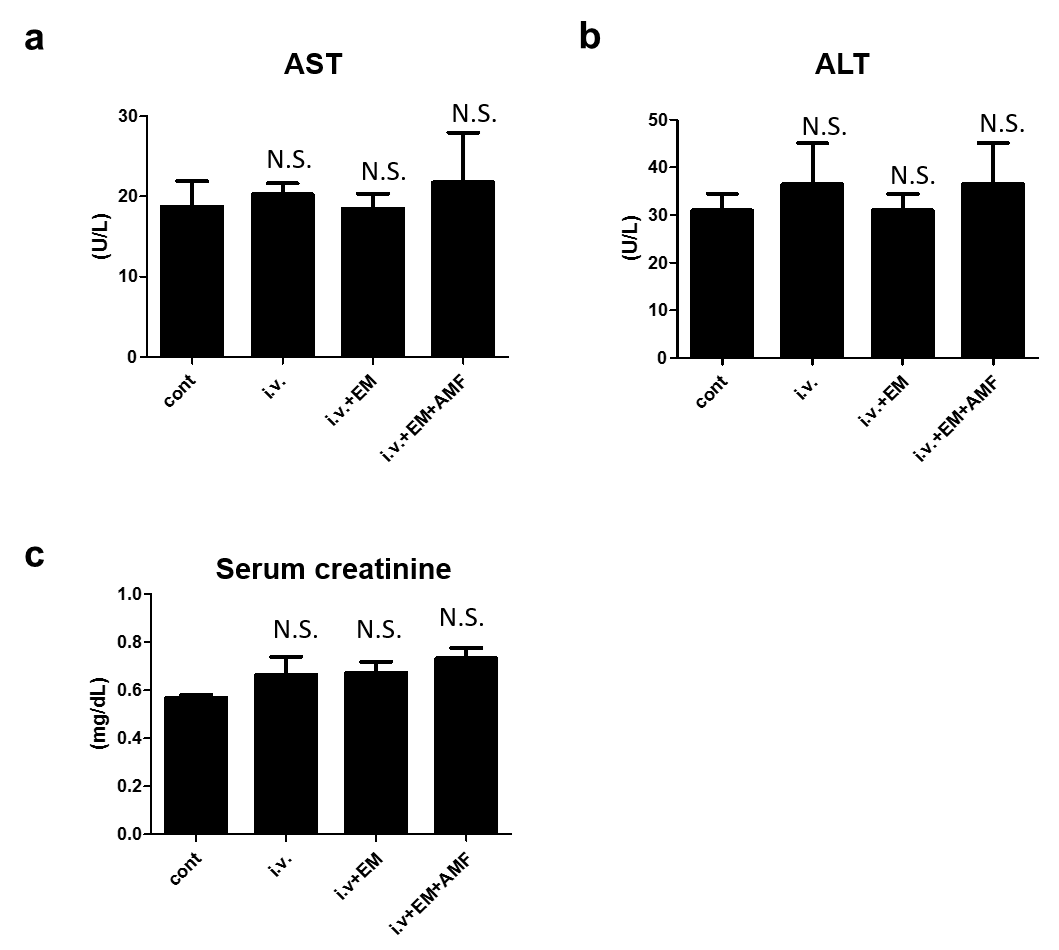
**

**Supplementary Figure 3. Liver and kidney function tests after Fe(Salen)** **nanoparticle injection/EM/AMF in rabbits.**

Seven days after intravenous injection of Fe(Salen) nanoparticles (5 mg/kg), or intravenous injection of Fe(Salen) nanoparticles (5 mg/kg) followed by electromagnet application (EM), or intravenous injection of Fe(Salen) nanoparticles (5 mg/kg) followed by EM/AMF (**Fig. 4**), serum AST (aspartate transaminase) , ALT (alanine transaminase) and creatinine concentrations were determined and compared with those of the untreated control. Note that there were no differences between the control and experimental groups (n=4, N.S., not significant)

**
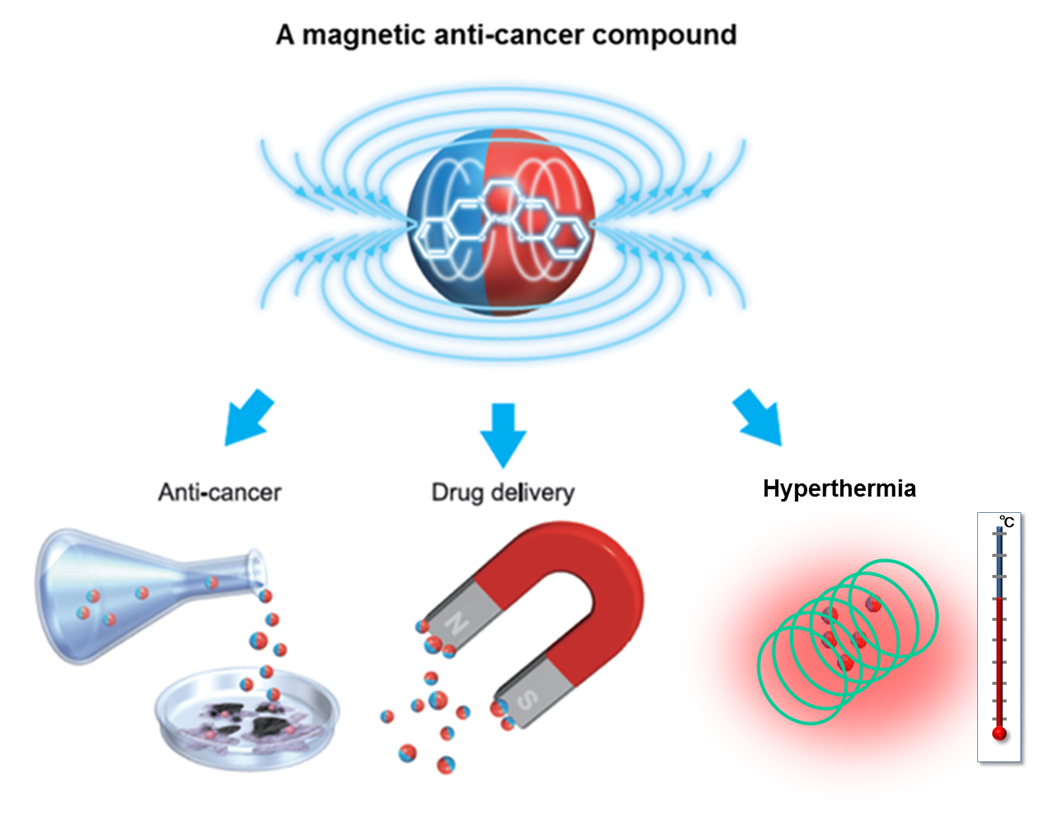
**

**Supplementary Figure 4. Schematic illustration of the use of Fe(II)salen complex for simultaneous hyperthermia-chemotherapy**

Because of its magnetic property, Fe(Salen) can be accumulated in cancer tissue in a controlled manner by the use of a magnet, and can also can produce heat upon exposure to an alternating magnetic field, thereby providing a basis for powerful, simultaneous anti-cancer hyperthermia-chemotherapy.
